# Supplementary material for: Structural transformation and the gender pay gap in Sub-Saharan Africa
Source: PLoS One. 2023 Apr 7;18(4):e0278188. doi: 10.1371/journal.pone.0278188 (PMC10081774; doi:10.1371/journal.pone.0278188)
Supplement: S1 Table — (DOCX) [file pone.0278188.s001.docx]

Table S1. Data cleaning of annual hours and earnings of non-farm employment in Malawi, Tanzania and Nigeria.

|  |  | **Malawi** | | **Tanzania** | | **Nigeria** | |
| --- | --- | --- | --- | --- | --- | --- | --- |
|  |  | # obs. | % | # obs. | % | # obs. | % |
| Annual hours | |  |  |  |  |  |  |
|  | >4368 hours | 5 | 0.09 | 167 | 4.88 | 62 | 1.62 |
|  | Missing | 10 | 0.17 | 164 | 4.80 | 170 | 4.43 |
| Annual earnings | |  |  |  |  |  |  |
|  | Assumption 6 days/week | 176 | 3.06 | 730 | 21.35 | 42 | 1.09 |
|  | Missing | 82 | 1.42 | 64 | 1.87 | 40 | 1.04 |
| Notes: Total number of non-farm employed people in the sample is 5,758 for Malawi; 3,419 for Tanzania; and 3,837 for Nigeria. | | | | | | | |
