# Supplementary material for: Structural transformation and the gender pay gap in Sub-Saharan Africa
Source: PLoS One. 2023 Apr 7;18(4):e0278188. doi: 10.1371/journal.pone.0278188 (PMC10081774; doi:10.1371/journal.pone.0278188)
Supplement: S2 Table — (DOCX) [file pone.0278188.s002.docx]

Table S2. Earnings functions of non-farm employment for men, women and pooled sample in rural Malawi, Tanzania and Nigeria with log hourly pay (real int. $) as dependent variable.

|  | **Malawi** | | | **Tanzania** | | | **Nigeria** | | |
| --- | --- | --- | --- | --- | --- | --- | --- | --- | --- |
|  | *Pooled* | *Women* | *Men* | *Pooled* | *Women* | *Men* | *Pooled* | *Women* | *Men* |
| Female | -0.069 |  |  | -0.019 |  |  | -0.173** |  |  |
|  | (0.135) |  |  | (0.090) |  |  | (0.072) |  |  |
| *Human capital* |  |  |  |  |  |  |  |  |  |
| Primary degree | -0.006 | 0.172 | -0.023 | 0.308*** | 0.259* | 0.326*** | 0.178 | 0.213 | 0.300 |
|  | (0.238) | (0.359) | (0.255) | (0.096) | (0.141) | (0.114) | (0.132) | (0.165) | (0.195) |
| Secondary degree | 0.596*** | 1.361*** | 0.326 | 0.674*** | 0.433 | 0.760*** | 0.415*** | 0.537*** | 0.587*** |
|  | (0.217) | (0.324) | (0.224) | (0.157) | (0.264) | (0.186) | (0.143) | (0.199) | (0.189) |
| Tertiary degree | 1.316*** | 1.525*** | 1.226*** | 1.767*** | 0.927* | 2.148*** | 0.798*** | 1.254*** | 0.741*** |
|  | (0.237) | (0.518) | (0.292) | (0.244) | (0.496) | (0.317) | (0.238) | (0.332) | (0.265) |
| Potential experience (years) | 0.011 | 0.009 | 0.028 | 0.021 | 0.053* | 0.010 | 0.024 | 0.062** | -0.012 |
|  | (0.024) | (0.037) | (0.034) | (0.020) | (0.029) | (0.030) | (0.018) | (0.026) | (0.022) |
| Square of experience | 0.000 | 0.000 | -0.001 | -0.000 | -0.001 | -0.000 | -0.000 | -0.001** | 0.001 |
|  | (0.000) | (0.001) | (0.001) | (0.000) | (0.001) | (0.001) | (0.000) | (0.000) | (0.000) |
| Multiple jobs | -0.199 | -0.011 | -0.324 | 0.151 | 0.402*** | -0.022 | -0.248*** | -0.032 | -0.586*** |
|  | (0.187) | (0.262) | (0.227) | (0.096) | (0.141) | (0.120) | (0.089) | (0.121) | (0.127) |
| *Sector* |  |  |  |  |  |  |  |  |  |
| Mining | -0.452 | -1.612 | -0.056 | 0.157 | 0.163 | 0.038 | 0.735*** | 1.003*** | 0.233 |
| *Ref. level: Commerce* | (0.395) | (1.743) | (0.435) | (0.383) | (0.513) | (0.420) | (0.252) | (0.353) | (0.492) |
| Manufacturing | -0.076 | -0.273 | 0.124 | -0.187 | -0.005 | -0.313* | 0.211 | 0.230 | 0.173 |
|  | (0.161) | (0.189) | (0.218) | (0.149) | (0.241) | (0.174) | (0.140) | (0.158) | (0.179) |
| Electricity, utilities | -0.112 | -0.079 | 0.041 | 0.378 | 0.536 | 0.416 | -0.043 | -0.085 | -0.283 |
|  | (0.465) | (0.947) | (0.531) | (0.358) | (0.335) | (0.364) | (0.321) | (0.373) | (0.331) |
| Construction | 0.648** | -0.047 | 0.732** | 0.521*** | 0.569 | 0.414** | 0.354 | -0.335 | 0.391* |
|  | (0.260) | (0.645) | (0.302) | (0.158) | (0.471) | (0.185) | (0.294) | (0.684) | (0.224) |
| Transport, storage, communication | 0.201 | 0.203 | 0.254 | 0.167 | 2.008*** | 0.061 | 0.213 | 0.634** | -0.122 |
|  | (0.437) | (0.683) | (0.491) | (0.239) | (0.342) | (0.259) | (0.225) | (0.286) | (0.244) |
| Finance, real estate | -0.330 | -0.825 | -0.156 | -0.638** |  | -0.741** | 0.359 | 0.665 | 0.238 |
|  | (0.839) | (0.575) | (0.979) | (0.264) |  | (0.317) | (0.322) | (0.712) | (0.434) |
| Other services | -0.274 | -0.591 | -0.226 | -0.061 | 0.005 | -0.068 | -0.030 | 0.137 | -0.265 |
|  | (0.207) | (0.398) | (0.258) | (0.139) | (0.167) | (0.206) | (0.115) | (0.127) | (0.164) |
| Missing sector | -0.650*** | -0.003 | -0.470 | 0.186 | 0.586*** | -0.027 | 0.086 | 0.673** | -0.085 |
|  | (0.228) | (0.331) | (0.315) | (0.123) | (0.190) | (0.145) | (0.338) | (0.332) | (0.358) |
| *Occupation* |  |  |  |  |  |  |  |  |  |
| Self-empl. with family labor | -0.075 | -0.112 | 0.151 | 0.166 | 0.137 | 0.022 | 0.327** | 0.325** | 0.389* |
| *Ref. level: Self-empl. without family labor* | (0.163) | (0.223) | (0.224) | (0.152) | (0.225) | (0.197) | (0.148) | (0.155) | (0.217) |
| Low-skilled employee | 1.146*** | 0.941** | 1.069*** | -0.116 | -0.469** | -0.063 | 1.084** | -0.058 | 1.667*** |
|  | (0.202) | (0.406) | (0.224) | (0.144) | (0.203) | (0.174) | (0.483) | (0.266) | (0.436) |
| Medium-skilled employee | 0.711*** | 1.315*** | 0.704*** | -0.214 | -0.367 | -0.173 | 1.015*** | 0.888*** | 0.908*** |
|  | (0.213) | (0.449) | (0.235) | (0.148) | (0.292) | (0.156) | (0.154) | (0.233) | (0.202) |
| High-skilled employee | 1.318*** | 1.805*** | 1.216*** | 0.791*** | 1.267*** | 0.479** | 1.439*** | 1.245*** | 1.503*** |
|  | (0.260) | (0.471) | (0.300) | (0.173) | (0.249) | (0.232) | (0.162) | (0.260) | (0.198) |
| Missing occupation | 1.759*** | 2.980** | 1.424*** | 0.865* | 1.307 | 0.652 | 1.281*** |  | 1.461*** |
|  | (0.458) | (1.209) | (0.346) | (0.510) | (0.939) | (0.469) | (0.257) |  | (0.215) |
| Constant | -1.233** | -0.263 | -2.180** | -0.906** | -2.023*** | -0.297 | -1.196*** | -1.801*** | -1.130** |
|  | (0.511) | (0.878) | (0.894) | (0.370) | (0.496) | (0.503) | (0.446) | (0.544) | (0.548) |
| Other controls | Y | Y | Y | Y | Y | Y | Y | Y | Y |
| Observations | 3,156 | 1,279 | 1,877 | 1,592 | 657 | 935 | 2,285 | 1,293 | 992 |
| R² | 0.131 | 0.213 | 0.125 | 0.152 | 0.220 | 0.148 | 0.229 | 0.205 | 0.266 |
| Notes: Population statistics are corrected using sampling weights. Significant coefficients are indicated with * p<0.1, ** p<0.05 and *** p<0.01 and standard errors are reported between parentheses. Other controls include dummies for region, proxy respondent, enumerator and month of interview. | | | | | | | | | |
