# Supplementary material for: Structural transformation and the gender pay gap in Sub-Saharan Africa
Source: PLoS One. 2023 Apr 7;18(4):e0278188. doi: 10.1371/journal.pone.0278188 (PMC10081774; doi:10.1371/journal.pone.0278188)
Supplement: S3 Table — (DOCX) [file pone.0278188.s003.docx]

Table S3. Earnings functions of non-farm employment for men, women and pooled sample in urban Malawi, Tanzania and Nigeria with log hourly pay (real int. $) as dependent variable.

|  | **Malawi** | | | **Tanzania** | | | **Nigeria** | | |
| --- | --- | --- | --- | --- | --- | --- | --- | --- | --- |
|  | *Pooled* | *Women* | *Men* | *Pooled* | *Women* | *Men* | *Pooled* | *Women* | *Men* |
| Female | -0.193* |  |  | -0.257*** |  |  | -0.063 |  |  |
|  | (0.100) |  |  | (0.068) |  |  | (0.069) |  |  |
| *Human capital* |  |  |  |  |  |  |  |  |  |
| Primary degree | 0.030 | 0.133 | -0.010 | 0.287*** | 0.382** | 0.192* | 0.181 | 0.387* | -0.143 |
|  | (0.153) | (0.200) | (0.175) | (0.086) | (0.148) | (0.107) | (0.183) | (0.226) | (0.286) |
| Secondary degree | 0.196 | 0.135 | 0.303* | 0.756*** | 0.912*** | 0.647*** | 0.575*** | 0.871*** | 0.207 |
|  | (0.127) | (0.197) | (0.156) | (0.126) | (0.213) | (0.151) | (0.213) | (0.252) | (0.307) |
| Tertiary degree | 1.176*** | 1.224*** | 1.176*** | 1.689*** | 1.755*** | 1.578*** | 1.205*** | 1.440*** | 0.796** |
|  | (0.131) | (0.225) | (0.166) | (0.222) | (0.354) | (0.236) | (0.267) | (0.319) | (0.348) |
| Potential experience (years) | 0.007 | 0.015 | 0.016 | 0.027 | 0.023 | 0.028 | 0.076*** | 0.075** | 0.082*** |
|  | (0.015) | (0.022) | (0.021) | (0.018) | (0.028) | (0.020) | (0.023) | (0.030) | (0.026) |
| Square of experience | -0.000 | -0.000 | -0.000 | -0.000 | -0.000 | -0.000 | -0.001** | -0.001* | -0.001*** |
|  | (0.000) | (0.000) | (0.000) | (0.000) | (0.001) | (0.000) | (0.000) | (0.001) | (0.001) |
| Multiple jobs | 0.001 | -0.098 | 0.050 | -0.153* | -0.078 | -0.243** | -0.297** | -0.048 | -0.560*** |
|  | (0.143) | (0.234) | (0.131) | (0.091) | (0.127) | (0.117) | (0.117) | (0.114) | (0.164) |
| *Sector* |  |  |  |  |  |  |  |  |  |
| Mining | -0.058 | -1.475** | 0.999** | 0.616*** | 0.888** | 0.399** | 0.698 | -0.382 | 0.978* |
| *Ref. level: Commerce* | (0.749) | (0.694) | (0.486) | (0.182) | (0.375) | (0.191) | (0.607) | (0.351) | (0.517) |
| Manufacturing | 0.149 | -0.113 | 0.335* | 0.018 | -0.102 | 0.100 | 0.235 | 0.242 | 0.255 |
|  | (0.150) | (0.187) | (0.181) | (0.128) | (0.210) | (0.128) | (0.175) | (0.240) | (0.166) |
| Electricity, utilities | 0.042 | -0.140 | 0.124 | -0.320 | -0.812*** | 0.256 | 0.123 | 0.096 | 0.264 |
|  | (0.271) | (0.729) | (0.256) | (0.369) | (0.247) | (0.332) | (0.328) | (0.379) | (0.274) |
| Construction | 0.711*** | 0.424 | 0.663*** | 0.571*** | 0.923* | 0.522*** | 0.685** | 0.716 | 0.623** |
|  | (0.197) | (0.614) | (0.205) | (0.111) | (0.494) | (0.127) | (0.314) | (0.582) | (0.288) |
| Transport, storage, communication | 0.320* | 0.990*** | 0.163 | 0.512*** | 1.219*** | 0.425*** | 0.485* | 0.495* | 0.363 |
|  | (0.166) | (0.303) | (0.176) | (0.129) | (0.440) | (0.134) | (0.250) | (0.287) | (0.299) |
| Finance, real estate | 1.083*** | 1.239** | 1.079*** | 0.334* | 0.634* | 0.143 | 0.319 | 0.383 | 0.297 |
|  | (0.283) | (0.554) | (0.214) | (0.189) | (0.323) | (0.214) | (0.218) | (0.333) | (0.294) |
| Other services | 0.204 | 0.426* | 0.174 | 0.172* | 0.260* | 0.141 | 0.066 | 0.147 | 0.051 |
|  | (0.163) | (0.249) | (0.169) | (0.099) | (0.144) | (0.136) | (0.169) | (0.205) | (0.210) |
| Missing sector | 0.257* | 0.214 | 0.359** | -0.286* | -0.008 | -0.419* | -0.055 | 0.084 |  |
|  | (0.152) | (0.281) | (0.164) | (0.165) | (0.146) | (0.221) | (0.339) | (0.435) |  |
| *Occupation* |  |  |  |  |  |  |  |  |  |
| Self-empl. with family labor | -0.414* | -0.323 | -0.735* | 0.201 | 0.040 | 0.342 | 0.441** | 0.536*** | 0.416 |
| *Ref. level: Self-empl. without family labor* | (0.216) | (0.202) | (0.413) | (0.171) | (0.236) | (0.213) | (0.179) | (0.194) | (0.279) |
| Low-skilled employee | 0.045 | -0.434 | 0.274 | -0.212* | -0.385** | -0.163 | 0.243 | -0.225 | 0.410* |
|  | (0.159) | (0.268) | (0.168) | (0.115) | (0.159) | (0.159) | (0.290) | (0.387) | (0.247) |
| Medium-skilled employee | 0.126 | -0.005 | 0.107 | -0.231*** | -0.415** | -0.151 | 0.515** | 0.287 | 0.559** |
|  | (0.126) | (0.255) | (0.136) | (0.085) | (0.168) | (0.099) | (0.211) | (0.281) | (0.244) |
| High-skilled employee | 0.833*** | 0.423 | 0.876*** | 0.405** | 0.237 | 0.520** | 0.970*** | 0.886*** | 1.043*** |
|  | (0.185) | (0.337) | (0.190) | (0.189) | (0.208) | (0.212) | (0.212) | (0.253) | (0.266) |
| Missing occupation | -0.037 | -0.709 | 0.118 | 0.285 | 0.159 | 0.392 | 0.823*** | 0.842*** | 0.874*** |
|  | (0.230) | (0.516) | (0.238) | (0.282) | (0.742) | (0.381) | (0.197) | (0.228) | (0.317) |
| Constant | -0.536 | -0.847 | -0.504 | -0.127 | -0.544 | 0.030 | -1.892*** | -2.449*** | -1.340** |
|  | (0.340) | (0.533) | (0.444) | (0.296) | (0.484) | (0.298) | (0.520) | (0.512) | (0.635) |
| Other controls | Y | Y | Y | Y | Y | Y | Y | Y | Y |
| Observations | 2,593 | 1,131 | 1,462 | 1,814 | 771 | 1,043 | 1,515 | 804 | 711 |
| R² | 0.221 | 0.235 | 0.254 | 0.222 | 0.234 | 0.234 | 0.236 | 0.207 | 0.277 |
| Notes: Population statistics are corrected using sampling weights. Significant coefficients are indicated with * p<0.1, ** p<0.05 and *** p<0.01 and standard errors are reported between parentheses. Other controls include dummies for region, proxy respondent, enumerator and month of interview. | | | | | | | | | |
