# Supplementary material for: Structural transformation and the gender pay gap in Sub-Saharan Africa
Source: PLoS One. 2023 Apr 7;18(4):e0278188. doi: 10.1371/journal.pone.0278188 (PMC10081774; doi:10.1371/journal.pone.0278188)
Supplement: S4 Table — (DOCX) [file pone.0278188.s004.docx]

Table S4. Probability of being non-farm employed for women and men aged 25-55 in rural Malawi, Tanzania and Nigeria.

|  | **Malawi** | | **Tanzania** | | **Nigeria** | |
| --- | --- | --- | --- | --- | --- | --- |
|  | *Women* | *Men* | *Women* | *Men* | *Women* | *Men* |
| Married | -0.011 | 0.216*** | -0.154*** | 0.027 | 0.134*** | 0.286*** |
|  | (0.017) | (0.031) | (0.024) | (0.029) | (0.032) | (0.033) |
| # children | -0.010* | -0.019* | -0.017** | -0.001 | 0.009 | 0.017*** |
|  | (0.005) | (0.011) | (0.007) | (0.008) | (0.007) | (0.006) |
| Landholdings (ha) | -0.044*** | -0.042** | -0.006* | -0.010** | 0.004 | -0.023** |
|  | (0.017) | (0.020) | (0.003) | (0.005) | (0.012) | (0.010) |
| Tropical livestock units | 0.022*** | 0.004 | -0.002 | -0.003* | -0.006** | -0.002 |
|  | (0.008) | (0.012) | (0.002) | (0.002) | (0.003) | (0.002) |
| Primary degree | 0.116*** | 0.080** | 0.092*** | 0.128*** | 0.163*** | 0.176*** |
|  | (0.025) | (0.032) | (0.024) | (0.027) | (0.029) | (0.031) |
| Secondary degree | 0.160*** | 0.209*** | 0.279*** | 0.399*** | 0.310*** | 0.304*** |
|  | (0.024) | (0.026) | (0.057) | (0.057) | (0.042) | (0.037) |
| Tertiary degree | 0.369*** | 0.512*** |  |  | 0.496*** | 0.398*** |
|  | (0.102) | (0.102) |  |  | (0.071) | (0.053) |
| Potential experience (years) | 0.007 | 0.003 | 0.009 | 0.032*** | 0.024*** | 0.017*** |
|  | (0.005) | (0.005) | (0.006) | (0.008) | (0.006) | (0.006) |
| Square of experience | -0.000* | -0.000 | -0.000* | -0.001*** | -0.000*** | -0.000** |
|  | (0.000) | (0.000) | (0.000) | (0.000) | (0.000) | (0.000) |
| Other controls | Y | Y | Y | Y | Y | Y |
| Observations | 7,509 | 6,530 | 2,334 | 2,072 | 3,147 | 2,210 |
| Pseudo R² | 0.081 | 0.088 | 0.077 | 0.081 | 0.090 | 0.149 |
| Adjusted Wald test | 4.67*** | 17.52*** | 16.35*** | 2.95** | 6.65*** | 26.81*** |
| Notes: Population statistics are corrected using sampling weights. Significant average marginal effects are indicated with * p<0.1, ** p<0.05 and *** p<0.01 and standard errors are reported between parentheses. Other controls include dummies for region, proxy respondent and month of interview. | | | | | | |
