# Supplementary material for: Structural transformation and the gender pay gap in Sub-Saharan Africa
Source: PLoS One. 2023 Apr 7;18(4):e0278188. doi: 10.1371/journal.pone.0278188 (PMC10081774; doi:10.1371/journal.pone.0278188)
Supplement: S5 Table — (DOCX) [file pone.0278188.s005.docx]

Table S5. Probability of being non-farm employed for women and men aged 25-55 in urban Malawi, Tanzania and Nigeria.

|  | **Malawi** | | **Tanzania** | | **Nigeria** | |
| --- | --- | --- | --- | --- | --- | --- |
|  | *Women* | *Men* | *Women* | *Men* | *Women* | *Men* |
| Married | -0.057 | 0.281*** | -0.210*** | 0.084*** | 0.134*** | 0.176*** |
|  | (0.037) | (0.044) | (0.026) | (0.025) | (0.036) | (0.027) |
| # children | -0.029* | -0.026** | -0.031** | -0.011 | 0.006 | 0.001 |
|  | (0.016) | (0.012) | (0.012) | (0.011) | (0.010) | (0.011) |
| Landholdings (ha) | -0.125** | 0.015 | -0.004 | -0.007 | -0.028 | -0.106*** |
|  | (0.049) | (0.040) | (0.005) | (0.005) | (0.027) | (0.033) |
| Tropical livestock units | -0.053 | -0.000 | -0.019** | -0.016*** | -0.003** | -0.043** |
|  | (0.036) | (0.013) | (0.008) | (0.004) | (0.002) | (0.020) |
| Primary degree | 0.025 | 0.085 | 0.110*** | 0.098*** | 0.255*** | 0.365*** |
|  | (0.039) | (0.071) | (0.039) | (0.033) | (0.050) | (0.040) |
| Secondary degree | 0.149*** | 0.221*** | 0.329*** | 0.154*** | 0.380*** | 0.385*** |
|  | (0.052) | (0.043) | (0.058) | (0.047) | (0.041) | (0.034) |
| Tertiary degree | 0.422*** | 0.348*** | 0.787*** | 0.198** | 0.334*** | 0.338*** |
|  | (0.061) | (0.065) | (0.168) | (0.096) | (0.069) | (0.043) |
| Potential experience (years) | 0.011 | 0.000 | 0.040*** | 0.007 | 0.022*** | 0.008 |
|  | (0.009) | (0.008) | (0.009) | (0.006) | (0.006) | (0.006) |
| Square of experience | -0.000 | 0.000 | -0.001*** | -0.000 | -0.000** | -0.000 |
|  | (0.000) | (0.000) | (0.000) | (0.000) | (0.000) | (0.000) |
| Other controls | Y | Y | Y | Y | Y | Y |
| Observations | 2,138 | 2,056 | 1,394 | 1,292 | 1,327 | 972 |
| Pseudo R² | 0.092 | 0.114 | 0.118 | 0.135 | 0.135 | 0.229 |
| Adjusted Wald test | 4.65*** | 13.27*** | 19.75*** | 9.52*** | 7.19*** | 11.97*** |
| Notes: Population statistics are corrected using sampling weights. Significant average marginal effects are indicated with * p<0.1, ** p<0.05 and *** p<0.01 and standard errors are reported between parentheses. Other controls include dummies for region, proxy respondent and month of interview. | | | | | | |
