# Supplementary material for: Structural transformation and the gender pay gap in Sub-Saharan Africa
Source: PLoS One. 2023 Apr 7;18(4):e0278188. doi: 10.1371/journal.pone.0278188 (PMC10081774; doi:10.1371/journal.pone.0278188)
Supplement: S7 Table — (DOCX) [file pone.0278188.s007.docx]

Table S7. Earnings functions of non-farm employment for men, women and pooled sample in urban Malawi, Tanzania and Nigeria with log hourly pay (real int. $) as dependent variable, corrected for selection bias using Heckman selection models.
