# Supplementary material for: Structural transformation and the gender pay gap in Sub-Saharan Africa
Source: PLoS One. 2023 Apr 7;18(4):e0278188. doi: 10.1371/journal.pone.0278188 (PMC10081774; doi:10.1371/journal.pone.0278188)
Supplement: S8 Table — (DOCX) [file pone.0278188.s008.docx]

Table S8. Kitagawa-Oaxaca-Blinder decomposition of gender pay gap for non-farm employed people aged 25-55 in Malawi, Tanzania and Nigeria, corrected for selection bias using Heckman selection models.

|  | **Malawi** | | **Tanzania** | | **Nigeria** | |
| --- | --- | --- | --- | --- | --- | --- |
|  | *Rural* | *Urban* | *Rural* | *Urban* | *Rural* | *Urban* |
| *Adjusted gender pay gap* | 3.876*** | -0.389* | 0.007 | 0.338* | 0.358** | 0.751*** |
|  | (0.587) | (0.204) | (0.364) | (0.201) | (0.172) | (0.143) |
| *A. Aggregate decomposition* |  |  |  |  |  |  |
| Endowment effect | 0.293*** | 0.188*** | 0.091** | 0.123*** | 0.399*** | 0.272*** |
|  | (0.060) | (0.059) | (0.047) | (0.037) | (0.054) | (0.059) |
| Endowment effect (share) | 7.56% | -48.38% | 1378.37% | 36.54% | 111.43% | 36.22% |
| Structural effect | 3.583*** | -0.578*** | -0.085 | 0.214 | -0.041 | 0.479*** |
|  | (0.581) | (0.200) | (0.359) | (0.201) | (0.178) | (0.133) |
| Structural effect (share) | 92.44% | 148.38% | -1278.37% | 63.46% | -11.43% | 63.78% |
| *B. Detailed decomposition* |  |  |  |  |  |  |
| *B1. Endowment effect* |  |  |  |  |  |  |
| Education (aggregated) | 0.116*** | 0.045 | 0.033** | 0.037** | 0.109*** | 0.123*** |
|  | (0.037) | (0.033) | (0.014) | (0.016) | (0.034) | (0.033) |
| Experience (aggregated) | -0.008 | 0.000 | -0.009 | 0.011 | 0.041** | 0.029* |
|  | (0.008) | (0.006) | (0.009) | (0.009) | (0.017) | (0.017) |
| Sector (aggregated) | -0.048 | 0.072** | 0.096*** | 0.095*** | 0.027 | 0.070** |
|  | (0.046) | (0.032) | (0.032) | (0.023) | (0.032) | (0.030) |
| Occupation (aggregated) | 0.245*** | 0.093** | -0.031 | -0.023 | 0.198*** | 0.064* |
|  | (0.059) | (0.044) | (0.035) | (0.023) | (0.032) | (0.034) |
| Context (aggregated) | -0.013 | -0.021 | 0.002 | 0.002 | 0.024 | -0.014 |
|  | (0.036) | (0.023) | (0.023) | (0.017) | (0.023) | (0.023) |
| *B2. Structural effect* |  |  |  |  |  |  |
| Education (aggregated) | 0.792*** | -0.049 | -0.342 | -0.031 | -0.103 | -0.304*** |
|  | (0.219) | (0.060) | (0.219) | (0.099) | (0.085) | (0.071) |
| Experience (aggregated) | -0.648 | 0.342 | -0.550 | -0.021 | -1.615*** | -1.045** |
|  | (0.757) | (0.521) | (0.589) | (0.395) | (0.601) | (0.446) |
| Sector (aggregated) | -0.268 | -0.325** | 0.349*** | 0.084 | 0.102 | -0.172* |
|  | (0.293) | (0.161) | (0.119) | (0.095) | (0.141) | (0.101) |
| Occupation (aggregated) | 0.460* | -0.224** | 0.156 | -0.105 | -0.496*** | -0.045 |
|  | (0.259) | (0.093) | (0.145) | (0.169) | (0.108) | (0.105) |
| Context (aggregated) | 0.669* | -0.406* | -0.181 | -0.114 | -0.466*** | 0.012 |
|  | (0.342) | (0.228) | (0.145) | (0.103) | (0.169) | (0.135) |
| Constant | 2.578** | 0.084 | 0.484 | 0.401 | 2.538*** | 2.033*** |
|  | (1.045) | (0.675) | (0.691) | (0.505) | (0.671) | (0.522) |
| Observations | 3,156 | 2,593 | 1,592 | 1,814 | 2,285 | 1,515 |
| Notes: Population statistics are corrected using sampling weights. Significant coefficients are indicated with * p<0.1, ** p<0.05 and *** p<0.01 and expressed in log-points. Standard errors are reported between parentheses. Selection models are presented in Tables S4 and S5, and adjusted earnings models in Tables S6 and S7. | | | | | | |
