# Supplementary material for: Structural transformation and the gender pay gap in Sub-Saharan Africa
Source: PLoS One. 2023 Apr 7;18(4):e0278188. doi: 10.1371/journal.pone.0278188 (PMC10081774; doi:10.1371/journal.pone.0278188)
Supplement: S9 Table — (DOCX) [file pone.0278188.s009.docx]

Table S9. Kitagawa-Oaxaca-Blinder decomposition of gender pay gap at the median for non-farm employed people aged 25-55 in Malawi, Tanzania and Nigeria.

|  | **Malawi** | | **Tanzania** | | **Nigeria** | |
| --- | --- | --- | --- | --- | --- | --- |
|  | *Rural* | *Urban* | *Rural* | *Urban* | *Rural* | *Urban* |
| *Median gender pay gap* | 0.575*** | 0.400*** | 0.010 | 0.307*** | 0.688*** | 0.348*** |
|  | (0.104) | (0.120) | (0.103) | (0.079) | (0.087) | (0.097) |
| *A. Aggregate decomposition* |  |  |  |  |  |  |
| Endowment effect | 0.289*** | 0.167** | 0.155*** | 0.093** | 0.477*** | 0.349*** |
|  | (0.065) | (0.074) | (0.054) | (0.041) | (0.060) | (0.061) |
| Endowment effect (share) | 50.22% | 41.87% | 1573.50% | 30.36% | 69.35% | 100.21% |
| Structural effect | 0.286*** | 0.232** | -0.146 | 0.214*** | 0.211*** | -0.001 |
|  | (0.104) | (0.104) | (0.097) | (0.074) | (0.080) | (0.090) |
| Structural effect (share) | 49.78% | 58.13% | -1473.50% | 69.64% | 30.65% | -0.21% |
| *B. Detailed decomposition* |  |  |  |  |  |  |
| *B1. Endowment effect* |  |  |  |  |  |  |
| Education (aggregated) | 0.126*** | 0.110*** | 0.029** | 0.027** | 0.105*** | 0.135*** |
|  | (0.032) | (0.039) | (0.013) | (0.013) | (0.039) | (0.034) |
| Experience (aggregated) | -0.006 | -0.002 | -0.008 | -0.002 | 0.035* | 0.034* |
|  | (0.010) | (0.008) | (0.010) | (0.008) | (0.018) | (0.019) |
| Sector (aggregated) | -0.073 | 0.045 | 0.149*** | 0.097*** | 0.091*** | 0.130*** |
|  | (0.048) | (0.041) | (0.040) | (0.025) | (0.031) | (0.032) |
| Occupation (aggregated) | 0.205*** | 0.041 | -0.018 | -0.037 | 0.217*** | 0.056* |
|  | (0.068) | (0.045) | (0.041) | (0.026) | (0.036) | (0.033) |
| Context (aggregated) | 0.036 | -0.026 | 0.003 | 0.008 | 0.029 | -0.005 |
|  | (0.038) | (0.029) | (0.023) | (0.018) | (0.023) | (0.021) |
| *B2. Structural effect* |  |  |  |  |  |  |
| Education (aggregated) | -0.076 | 0.069 | -0.129 | 0.019 | -0.005 | -0.142** |
|  | (0.150) | (0.052) | (0.188) | (0.090) | (0.087) | (0.061) |
| Experience (aggregated) | 0.083 | -0.333 | -0.960 | -0.029 | -0.698 | -0.108 |
|  | (0.673) | (0.375) | (0.637) | (0.417) | (0.605) | (0.464) |
| Sector (aggregated) | 0.065 | -0.367** | 0.443*** | 0.201* | 0.502*** | -0.429** |
|  | (0.273) | (0.143) | (0.145) | (0.110) | (0.190) | (0.183) |
| Occupation (aggregated) | 0.156 | -0.248** | -0.002 | -0.202 | -0.729*** | -0.136 |
|  | (0.219) | (0.117) | (0.151) | (0.167) | (0.159) | (0.170) |
| Context (aggregated) | 0.340 | -0.096 | -0.961*** | -0.211* | 0.016 | -0.143 |
|  | (0.246) | (0.192) | (0.370) | (0.126) | (0.148) | (0.139) |
| Constant | -0.282 | 1.208*** | 1.463** | 0.436 | 1.124* | 0.957* |
|  | (0.794) | (0.456) | (0.718) | (0.454) | (0.655) | (0.567) |
| Observations | 3,156 | 2,593 | 1,592 | 1,814 | 2,285 | 1,515 |
| Notes: Population statistics are corrected using sampling weights. Significant coefficients are indicated with * p<0.1, ** p<0.05 and *** p<0.01 and expressed in log-points. Standard errors are reported between parentheses. | | | | | | |
