# Supplementary material for: Structural transformation and the gender pay gap in Sub-Saharan Africa
Source: PLoS One. 2023 Apr 7;18(4):e0278188. doi: 10.1371/journal.pone.0278188 (PMC10081774; doi:10.1371/journal.pone.0278188)
Supplement: Attachment — Submitted filename: PlosOne_response letter.docx [file pone.0278188.s016.docx]

Dear Editor and Reviewers,

We thank you for your very constructive comments on the previous version of the paper. These comments helped us to improve the paper by restructuring the organization of the text and tables, better articulating the motivation behind our strategy and improving our methods. Please find below our point-by-point responses (in blue), in which we explain in detail how we addressed all your comments.

**Editor:**

When submitting your revision, we need you to address these additional requirements.

1. Please ensure that your manuscript meets PLOS ONE's style requirements, including those for file naming. The PLOS ONE style templates can be found at

[https://journals.plos.org/plosone/s/file?id=wjVg/PLOSOne_formatting_sample_main_body.pdf](https://eur03.safelinks.protection.outlook.com/?url=https%3A%2F%2Fjournals.plos.org%2Fplosone%2Fs%2Ffile%3Fid%3DwjVg%2FPLOSOne_formatting_sample_main_body.pdf&data=04%7C01%7Cgoedele.vandenbroeck%40uclouvain.be%7C9297bcae48744c95713f08d9f22c07bc%7C7ab090d4fa2e4ecfbc7c4127b4d582ec%7C0%7C0%7C637807094397929097%7CUnknown%7CTWFpbGZsb3d8eyJWIjoiMC4wLjAwMDAiLCJQIjoiV2luMzIiLCJBTiI6Ik1haWwiLCJXVCI6Mn0%3D%7C2000&sdata=PGZrJqw8bbs8CW4SL1v9BNF1pMqqKBqd20x5RRofmxA%3D&reserved=0)  and

[https://journals.plos.org/plosone/s/file?id=ba62/PLOSOne_formatting_sample_title_authors_affiliations.pdf](https://eur03.safelinks.protection.outlook.com/?url=https%3A%2F%2Fjournals.plos.org%2Fplosone%2Fs%2Ffile%3Fid%3Dba62%2FPLOSOne_formatting_sample_title_authors_affiliations.pdf&data=04%7C01%7Cgoedele.vandenbroeck%40uclouvain.be%7C9297bcae48744c95713f08d9f22c07bc%7C7ab090d4fa2e4ecfbc7c4127b4d582ec%7C0%7C0%7C637807094397929097%7CUnknown%7CTWFpbGZsb3d8eyJWIjoiMC4wLjAwMDAiLCJQIjoiV2luMzIiLCJBTiI6Ik1haWwiLCJXVCI6Mn0%3D%7C2000&sdata=GiPCyaOQBz6vezW3GgKJGEdMS0iAKg9LCkJPRFp1ixk%3D&reserved=0)

**Response:** We re-checked the requirements and made sure that our manuscript meets them.

2. Please amend your current ethics statement to address the following concerns:

a) Did participants provide their written or verbal informed consent to participate in this study?

b) If consent was verbal, please explain i) why written consent was not obtained, ii) how you documented participant consent, and iii) whether the ethics committees/IRB approved this consent procedure.

**Response:** The new ethics statement is as follows: “The surveys were implemented by the respective national statistical office (NSO) in Malawi, Nigeria and Tanzania. Each survey was implemented under the respective Statistical Act of that country, which governs the roles, responsibilities, and mandates of the respective NSO in producing and disseminating official statistics that each survey informed. Per the powers granted to the NSOs in fulfilling their duties, the NSO-implemented surveys are not subjective to approval by an Institutional Review Board (IRB) approval. However, per the established practice followed by the respective NSO in each country, the survey participants did provide informed consent prior to the face-to-face interview.”

3. Thank you for stating the following in the Acknowledgments Section of your manuscript:

“This research was financially supported by the Research Foundation Flanders (FWO) (FWO post-doctoral fellowship).”

We note that you have provided additional information within the Acknowledgements Section that is not currently declared in your Funding Statement. Please note that funding information should not appear in the Acknowledgments section or other areas of your manuscript. We will only publish funding information present in the Funding Statement section of the online submission form.

Please remove any funding-related text from the manuscript and let us know how you would like to update your Funding Statement. Currently, your Funding Statement reads as follows:

“G.V.d.B. was financially supported by the Research Foundation Flanders (FWO) (FWO post-doctoral fellowship). The funders had no role in study design, data collection and analysis, decision to publish, or preparation of the manuscript.”

Please include your amended statements within your cover letter; we will change the online submission form on your behalf.

**Response:** We changed the Acknowledgments in the manuscript to: “We thank seminar participants at Wageningen University & Research, UCLouvain and KULeuven for their feedback, and are grateful for the comments provided by two anonymous reviewers.”

The funding statement can remain as it was in the original version.

**Reviewers' comments:**

**Reviewer #1:**

The study is well motivated, the approach followed is appropriate and the data base used is very informative. The paper and the results obtained are interesting for the potential reader.
Having said this, I think the article would benefit from a re-ordering of the sections and tables since structure is a bit messy.

I do not understand why Tables 1 and 2 are in the main text and the rest of tables are in Supplementary Annexes. What is the relevance of Table 1 to be in the main text? It seems to me that the most important table is S10 (in fact, the two figures are derived from that table, which captures the results of the Oaxaca-Blinder decomposition). Tables S1 and S3 are auxiliary; Table S2 seems to be useless (unless authors provide some convincing arguments); Tables S4 and S5 on selection into non-farm employment as robustness checks are presented even before any estimation is carried out; descriptive tables S6 and S7 are presented after regressions; and so on….

In my opinion, a more sensible organization of the paper would be something similar to this:
1. Introduction---
2. Materials and methods (Data, Measurement of hourly pay, and perhaps decomposition analysis---
3. Characteristics of NF-E, and Data description with Tables S6 and S7 (maybe Table 1 if authors consider it)
4. The decomposition analysis with Tables S8 and S9 (may be in Annexes) to present the wage regressions, and then Table 2 and Table S10.

[Aside] Authors should notice that data in Table 2 are sample data and in S10 are estimated. It makes a bit difficult to interpret the mean pays in Table S10 as when compared with table 2. If Average rural pay is 2.61 for men and 1.67 for women, then the difference is 0.94; could not be easier to split that difference between explained and not explained (something like 0.62 explained and 0.32 unexplained)? It’s a bit messy to compute exp(0,528), obtain 1,70, and then saying that women earn 70% less than men.

5. Now, the Robustness checks with results of Table S13 (tables S4, S5, S11 and S12 can still be in the Annexes).

**Response:** Thank you for valuing the quality of our work and for suggesting how to improve the organization of the paper. We have restructured the paper according to your suggestions. In addition, we dropped tables 2, S1 and S2 and moved tables S6, S7 and S10 to the main text (now tables 2, 3 and 4).

Other comments
Some questions should be addressed in the study
1. Why the choice of these three countries? Data availability?, different stage in structural transformation?

**Response:** We now motivate this better in the Introduction (lines 345-363):

“The choice for including Malawi, Nigeria and Tanzania in our analysis is partly motivated by their different stage in structural transformation and partly based on data availability. First, according to several World Development Indicators [39], Nigeria tends to be at a more advanced stage of structural transformation than Tanzania, followed by Malawi. For example, gross domestic product (GDP) per capita in 2019 was 5,348 int.$ in Nigeria, 2,771 int.$ in Tanzania and 1,104 int.$ in Malawi. Non-agricultural employment accounted for a higher share of total employment in Nigeria (65%), compared to Tanzania (35%) and Malawi (24%). Also, the urbanization rate in 2019 was highest in Nigeria (51%), followed by Tanzania (35%) and Malawi (17%). All three countries have embarked on the path of structural transformation during the past 20 years, albeit at different paces with growth rates highest in Nigeria and lowest in Malawi.

Second, we leverage household survey data that are representative at the national-level and for urban and rural areas, and that cover extensive, individual-level information on labor earnings and inputs, including wage- and self-employment activities. Such data are in general quite rare for Sub-Saharan Africa, but data collection efforts have steadily improved during the past ten years, both in terms of quantity and quality. An important initiative has been the World Bank Living Standards Measurement Study – Integrated Surveys on Agriculture (LSMS-ISA) project, which has provided technical and financial support for multi-topic, national and longitudinal household surveys that are conducted by national statistical offices in select African countries. While the LSMS-ISA covers multiple countries, the specific data requirements for the purpose of our study are only fulfilled by the data from Malawi, Nigeria and Tanzania.”

2. Is different stage in structural transformation granted only by different GDP growth and urbanization rate? One would need more reasons to argue that these countries are in different stages in structural transformation ….one doesn’t know the starting point

**Response:** As described above, we provide more statistics from the World Development Indicators database to show that these three countries are in different stages in structural transformation, and how they have evolved during the past 20 years.

3. In the robustness checks the authors control for selection into NF-Employment but not into non-participation. Would it be important to control for this?

**Response:** Our selection correction accounts for bias associated with selection into the estimation sample, and includes all women and men age 25-55, i.e., those in non-farm employment, those in farm employment, and those not in any employment. Hence, farm workers and the inactive population are all included in the selection model, so when we account for selection into non-farm employment this includes selection into any employment. We clarify this in the section on Robustness checks (lines 580-583):

“First, we use Heckman selection models to control for non-random selection into non-farm employment. In the first stage, we use probit models to estimate the probability of being non-farm employed, for the entire sample of individuals age 25-55 (including those not employed and those working in farming).”

In addition, and as further described in response to your next comment (#4), we analyze a second robustness check based on recentered influence functions to decompose the gender pay gap at the median, which is less susceptible to non-random selection into our estimation sample. Hence this also accounts for selection into any employment as well as into non-farm employment.

Finally, we added more information in the section on “Measurement of hourly pay” to explain why we focus on non-farm employment, as opposed to overall employment (lines 413-424):

“We focus specifically on non-farm employment, as opposed to overall employment, for the main reason that individual earnings tied to household farms are very hard (if not impossible) to estimate. For instance, the survey questions to elicit data on revenues and costs are typically asked at different levels: while the questions on costs of pesticides, fertilizer and labor are asked at the plot-level, the information regarding costs of seeds and revenues from market sales are elicited at the crop-level, and the data on rental/depreciating costs of farm equipment are collected at the farm-level. Arguably more challenging is the allocation of net household agricultural income across household laborers, as it would be highly sensitive to the choice of the survey questions that are used for this purpose (e.g., questions that capture owners versus managers versus laborers at the plot- and livestock group-level). In addition, a lot of the work done in farm employment is unpaid, with the resulting production destined for own consumption, which is especially the case for women’s work [40] and further complicates the estimation of individual earnings and gender pay gaps.”

4. Regarding the choice of selection variables, it is said that variables like marital status and number of children affect employment but not hourly pay. There is various evidence showing that married men earn more than married women, and having children reduce earnings of women. Authors should rely in other variables as a choice for controlling selection (in fact, the authors find that the lambda term is non-significant).

**Response:** We acknowledge that our selection variables may not be perfectly exogenous to hourly pay. However, these are the best suitable variables that we could identify among the available data across the three countries. In the revised paper, we rewrote the section on “Robustness checks”, to give a more nuanced interpretation of the results from this Heckman correction and to include a more extensive description of a second robustness check based on recentered influence functions. This involves decomposing the gender pay gap at the median, which is less susceptible to non-random participation and an arbitrary choice of selection variables (lines 581-606):

“In the first stage, we use probit models to estimate the probability of being non-farm employed. We control for education, experience and survey settings. We include marital status, number of children, landholdings and livestock units as selection variables. Theory and other studies suggest that these are major determinants of non-farm employment status but are less likely to influence hourly pay directly [42]. Adjusted Wald tests reject the null hypothesis that these selection variables can jointly be excluded in all the regression models, indicating that they are good predictors of participation in non-farm employment (S4-5 Tables). We obtain qualitatively similar results when only marital status and landholdings, the strongest predictors for non-farm employment, are used as selection variables. In the second stage, we decompose the gender pay gap using the adjusted earnings functions. We use maximum likelihood estimations and take into account the household survey design. We find that selection bias, as indicated by ρ in Tables S6-7, is either insignificant in rural and urban Malawi and Tanzania or moves in the same direction for both men and women in rural Nigeria, and hence does not influence the estimated gender pay gap. We do find a negative selection bias for rural men in Nigeria, leading to a smaller adjusted pay gap of 43 percent compared to 77 percent in our main results. We therefore conclude that most of our results are robust to non-random participation in non-farm employment.

Our selection variables may not be perfectly exogenous to hourly pay estimates. Thus, as a second robustness check, we analyze recentered influence functions to decompose the gender pay gap at the median [43]. Median values are less susceptible to skewed distributions. Moreover, if selection into non-farm employment is non-random, recentered influence functions only require assumptions on the position of the missing pay observations with respect to the median, rather than assumptions on the exogeneity of selection variables [44]. We do not find statistically significant differences between the average and median pay gap and their decompositions (S9 Table), again suggesting that our estimates are robust.”

5. From data in Table 1, one deduces that farm-employment in rural Malawi and Tanzania is about 50%, which are not considered in the study. It may affect to differences in pay in a marked way.

**Response:** As explained in our response to comment 3, we focus specifically on non-farm employment in our study due to challenges related to measuring individual earnings from farm work. We dropped overall employment rates from Table 1 to avoid confusion. In our Discussion section, we do compare our non-farm gender pay gap estimates to findings from studies on agricultural productivity differentials in the same countries to discuss what a shift from farm to non-farm employment could entail (lines 663-669):

“Structural change within rural areas also entails a shift from farm to non-farm activities. In view of the existing estimates of the productivity differences on female-managed and male-managed agricultural plots of 25 percent in Malawi [33], 35 percent in North Nigeria [34] and 29 percent in Tanzania [35], we conclude that this shift implies a reduction of the gender pay gap in Tanzania, but an increase in Malawi and (especially) Nigeria. While the rural non-farm economy has often been promoted to diversify income sources and reduce poverty, our results point to the need to consider gender-sensitive policies in this shift to ensure that women benefit equally from improvements in overall welfare.”

6. Why survey recall period of 12 months is the main specification (line 215) instead of 7 days (which is usual in Europe)?

This is because of seasonality effects, which are more prevalent in Africa than in Europe. We added a sentence in the section “Measurement of hourly pay” to explain this (lines 412-413): “Participation in non-farm employment is based on the 12 months prior to the survey to avoid seasonality effects that are common in these countries.”

In addition, as is clear from Table 1, non-farm employment rates are similar between the 7-day recall period and the 12-month recall period.


Minor comments:
- References are needed for the assertion in lines 53-56

We added the following key reference:

C. Olivetti, B. Petrongolo, The economic consequences of family policies: lessons from a century of legislation in high-income countries. *J Econ Perspect* *31*(1), 205-30 (2017).

- Why is not explicitly cited the seminar work by Oaxaca (1979) and Blinder (1979) (lines 104-9, 189-97)?

We added following references to the Introduction and Methods, and now refer explicitly to the Kitagawa-Oaxaca-Blinder decomposition:

1. Kitagawa, E. M. (1955). Components of a difference between two rates. *Journal of the American statistical association*, *50* (272), 1168-1194.
2. Oaxaca, R. (1973). Male-female wage differentials in urban labor markets. *International economic review*, 693-709.
3. Blinder, A. S. (1973). Wage discrimination: reduced form and structural estimates. *Journal of Human resources*, 436-455.

We include the reference to Kitagawa (1955), which may not be well known to many economists, as it recently came to our attention that her 1955 paper was the first publication on the method.

-Line 257 it lacks “and only for 50% in urban Malawi”.

We rephrased this and the sentence is now as follows (lines 530-532):

“The variation in the endowment effect is much wider across urban areas, where differences in characteristics account for only 32 percent of the pay gap in Tanzania, 50 percent in Malawi, and 81 percent in Nigeria.”

**Reviewer #2:**

The paper titled "Structural transformation and the gender pay gap in Sub-Saharan Africa" analyzes the gender pay gap in Malawi, Tanzania and Nigeria in recent years and relate it with the structural transformation of the labour market and progressive urbanization of the region. The paper is well written, the methodology applied is standard but mostly well executed (see comments below), and the analysis of the gender pay gap in less developed countries and its evolution along their development path is certainly a topic that deserves attention.

To me there is one important question to be clarified, that is the way in which the link between structural transformation and gender pay gap is analyzed. The paper claims that the three countries under investigation are on different level of structural transformation, so analyzing differences between countries can give information about how the structural transformation is related with the evolution of gender pay gap. However, given the many substantial differences between these three countries, claiming that differences in the pay gap may be explained by the different level of structural information is not reliable. The alternative to compare urban vs rural areas also is not actually indicative of the structural transformation process alone, also because the definition of rural vs urban areas is not fully consistent in between countries. So I would be more careful in interpreting the results in terms of structural transformation.

To improve the analysis of the aforementioned link, one possible empirical strategy could be that the gender pay gap in each country was analyzed at different points in time. This is certainly possible for Malawi as similar data is available since the late nineties. This could be done to a lesser extent in Nigeria, although similar data started only in 2006 (if I remember well), unsure for Tanzania. The authors could decide to focus just on one country or two, depending on data availability and relevance of the structural transformation progress.

**Response:** We thank the reviewer for acknowledging the importance of our work and for suggesting an alternative empirical approach that could leverage additional rounds of data in at least Malawi and Nigeria. Unfortunately, various data issues do not allow us to explore the latter suggestion. The earlier rounds of the Malawi Integrated Household Survey (IHS) do not use comparable survey questionnaire modules on labor and non-farm enterprises, vis-à-vis the later rounds that received technical assistance under the World Bank LSMS-ISA initiative, beginning in 2010/11. In the case of Nigeria, we have a related comparability issue, in the sense that while the Nigeria Living Standards Survey (NLSS) has rounds prior to the General Household Survey – Panel (i.e., the source of our survey data in Nigeria), the data from these two surveys are not based on comparable questionnaire modules. To better nuance how we investigate possible linkages between structural transformation and gender pay gap, we made several changes throughout the paper.

First, we rewrote parts of our Introduction to motivate our strategy better (lines 329-344):

“To help fill knowledge gaps, we first provide evidence on the extent and drivers of the gender pay gap in non-farm wage- and self-employment activities in Malawi, Nigeria and Tanzania. We then describe linkages between the gender pay gap and structural transformation through a spatial comparison across and within these three countries. Ideally, structural transformation is investigated by analyzing countries at different points in time to capture the development process in the long run. However, this requires extensive longitudinal surveys with consistent high-quality data on individual earnings from wage- and self-employment in both urban and rural areas. Such data are not yet available for Sub-Saharan Africa. Therefore, we opt for a different strategy: we do not only compare countries that are at a different stage in structural transformation, but also compare rural and urban areas in each country. We apply the Kitagawa-Oaxaca-Blinder decomposition method that has been widely used in labor economics to decompose the gender pay gap into (i) a component that is explained by gender differences in observable characteristics, such as education, sector, and occupation, and (ii) another component that is explained by gender differences in returns to these characteristics [36-38]. We pay specific attention to education, sector and occupation, as structural transformation is typically associated with changes in these dimensions. On the whole, we provide important insights into patterns that are common to multiple countries, but also reveal country-specific findings.”

Second, we also motivate our choice of countries better in the Introduction (lines 345-363):

“The choice for including Malawi, Nigeria and Tanzania in our analysis is partly motivated by their different stage in structural transformation and partly based on data availability. First, according to several World Development Indicators [39], Nigeria’s economy is at a more advanced stage of structural transformation than Tanzania, followed by Malawi. For example, gross domestic product (GDP) per capita in 2019 was 5,348 int.$ in Nigeria, 2,771 int.$ in Tanzania and 1,104 int.$ in Malawi. Non-agricultural employment accounted for a higher share of total employment in Nigeria (65%), compared to Tanzania (35%) and Malawi (24%). Also, the urbanization rate in 2019 was highest in Nigeria (51%), followed by Tanzania (35%) and Malawi (17%). All three countries have embarked on the path of structural transformation during the past 20 years, albeit at different paces with growth rates highest in Nigeria and lowest in Malawi.

Second, we leverage household survey data that are representative at the national-level and for urban and rural areas, and that cover extensive, individual-level information on labor earnings and inputs, including wage- and self-employment activities. Such data are in general quite rare for Sub-Saharan Africa, but data collection efforts have steadily improved during the past ten years, both in terms of quantity and quality. An important initiative has been the World Bank Living Standards Measurement Study - Integrated Surveys on Agriculture (LSMS-ISA) project, which has provided technical and financial support for multi-topic, national and longitudinal household surveys that are conducted by national statistical offices in select African countries. While the LSMS-ISA covers multiple countries, the specific data requirements for the purpose of our study are only fulfilled by the data from Malawi, Nigeria and Tanzania.”

In addition, we now explain in the Data section why we use these specific survey waves (lines 370-383):

“We use these specific waves as they were the most recent available at the time of the analysis. For Tanzania, more recent data (for 2014/15) was available but not used, because data on annual hours were missing for 39% of workers (the self-employed). In the meantime, more recent waves have become available (Malawi Fifth Integrated Household Survey 2019/20, Nigeria General Household Survey – Panel 2018/19, and Tanzania National Panel Survey 2019/20). While it is possible to add these waves as additional data points in our analysis, the time between surveys is still limited (at most 7 years in the case of Tanzania). Over this time span, labor force participation rates were virtually constant and the agricultural share of employment declined only slightly, according to the ILO modelled estimates. Hence, we would not be getting much additional insight into the link between structural change and the gender wage gap. Finally, we initially considered other countries that are included in the LSMS-ISA initiative as well, such as Ethiopia, Uganda, Niger, Mali and Burkina Faso. However, we found that the quality of data on earnings and inputs in non-farm employment was not sufficient for these countries, due to missing individual-level information for self-employment or no distinction between wage and self-employment.”

Other Empirical points.
1) For all three countries more recent data are available. It would be a nice plus to update the results to the latest available data.

**Response:** As described above, updating the results to the latest available data is unlikely to affect our findings, given the short time span between the data we analyze and the more recent data available, and the limited changes in labor force participation and agricultural employment over that period. Updating the results would not be a trivial task, given the challenges involved in data cleaning and the construction of comparable labor variables. We therefore decided not to pursue this update, but we motivate our choice for the specific survey waves more clearly in the Data section.

2) It seems that the results for the pooled sample are mostly driven by Nigeria, which is not surprising given the population difference. That said, and given the concerns about differences between countries, the paper would be equally interesting without presenting the results for the pooled sample.

**Response:** We agree with this suggestion, we removed the pooled sample results from all tables and now focus exclusively on the country-specific results, and their comparison.

3) Hourly earnings may be a quite volatile measure, for instance for self-employment it could be especially low because the declared work hours is generally very large. Although it is quite standard for developed countries, I wonder whether for a developing country using weekly earnings and controlling for the working hours for the decomposition could be an option? That could also allow to avoid winsorizing the distribution.

**Response:** It is indeed the case that part of the variation in hourly earnings will come from differences in hours worked and these hours can be very high, especially for the self-employed. However, we believe this is relevant variation, in the sense that hourly earnings captures the returns to one’s labor. If we focus instead on weekly earnings, our measure of the gender gap will conflate differences in work hours (which are likely to vary across sectors and occupations as well) with differences in wages. Furthermore, the use of hourly earnings allows us to compare our gender pay gap estimates with those for high-income countries.

Still, to reduce effects of outlier values for hourly earnings on our gender pay gap estimates, we also decompose the gender pay gap at the median. We describe this as our second robustness check in the revised section on Robustness checks (lines 598-606), as also described in our answer to the next comment).

4) For the sample selection model, the choice of the selection variables seems rather unusual. Besides marital status, which clearly influence the work status (besides being in non-agricultural work), the others are all variables that depend on the choice of being in agricultural job or not. For instance, it is more important for agricultural workers to have a large number of children. Of course having landholding and livestock are clearly influenced by the choice of being in agricultural work (ex post). Then other exclusion restrictions would be needed to make the sample selection correction more credible.

**Response**: We acknowledge that our selection variables are unlikely to be perfectly exogenous to hourly pay. However, these are the most suitable variables available in the three datasets, and they have been frequently used in other studies (perhaps also due to lack of better alternatives). To reflect this concern with the selection variables, we nuance the interpretation of results from this model more clearly in the revised section on Robustness checks. In addition, we include a more extensive description of a second robustness check based on recentered influence functions. This involves decomposing the gender pay gap at the median, which is less susceptible to non-random participation and an arbitrary choice of selection variables (lines 581-606):

“In the first stage, we use probit models to estimate the probability of being non-farm employed. We control for education, experience and survey settings. We include marital status, number of children, landholdings and livestock units as selection variables. Theory and other studies suggest that these are major determinants of non-farm employment status but are less likely to influence hourly pay directly [42]. Adjusted Wald tests reject the null hypothesis that these selection variables can jointly be excluded in all the regression models, indicating that they are good predictors of participation in non-farm employment (S4-5 Tables). We obtain qualitatively similar results when only marital status and landholdings, the strongest predictors for non-farm employment, are used as selection variables. In the second stage, we decompose the gender pay gap using the adjusted earnings functions. We use maximum likelihood estimations and take into account the household survey design. We find that selection bias, as indicated by ρ in Tables S6-7, is either insignificant in rural and urban Malawi and Tanzania or moves in the same direction for both men and women in rural Nigeria, and hence does not influence the estimated gender pay gap. We do find a negative selection bias for rural men in Nigeria, leading to a smaller adjusted pay gap of 43 percent compared to 77 percent in our main results. We therefore conclude that most of our results are robust to non-random participation in non-farm employment.

Our selection variables may not be perfectly exogenous to hourly pay estimates. Thus, as a second robustness check, we analyze recentered influence functions to decompose the gender pay gap at the median [43]. Median values are less susceptible to skewed distributions. Moreover, if selection into non-farm employment is non-random, recentered influence functions only require assumptions on the position of the missing pay observations with respect to the median, rather than assumptions on the exogeneity of selection variables [44]. We do not find statistically significant differences between the average and median pay gap and their decompositions (S9 Table), again suggesting that our estimates are robust.”
