# Supplementary material for: Structural transformation and the gender pay gap in Sub-Saharan Africa
Source: PLoS One. 2023 Apr 7;18(4):e0278188. doi: 10.1371/journal.pone.0278188 (PMC10081774; doi:10.1371/journal.pone.0278188)
Supplement: Attachment — Submitted filename: Response letter_R2.docx [file pone.0278188.s017.docx]

**Response letter for PONE-D-21-36418R1:**

**“Structural transformation and the gender pay gap in Sub-Saharan Africa”**

**Reviewer #1:**

I think the article has benefited from the reordering, now being much easier to read, understand and follow. Most of the issues I pointed out in the first review have been satisfactorily addressed by the authors. However, in my opinion there are still two questions whose answers have not completely satisfied me, and I would like to ask for some additional clarification.

**Response:** Thank you for your additional feedback on our article. We explain below point-by-point how we addressed your comments.

First, the authors claim that countries are at different stages of structural transformation according to indicators on GDP per capita, non-agricultural employment, and urbanization rates. Indeed, based on these data, it appears that Nigeria is "ahead" of Tanzania and Malawi. However, we do not know the starting point of each of the countries. We do not know how they were in the year 2000, which is the date that the authors consider the beginning of the structural transformation. The authors claim that (page 5, lines 353-354), “All three countries have embarked on the path of structural transformation during the past 20 years, albeit at different paces with growth rates highest in Nigeria and lowest in Malawi.” One can think that the countries were in a similar situation in year 2000 and that Nigeria has grown much faster. However, it is most likely that Nigeria was already the most advanced country in 2000. It is not clear from what is said in the text, and this may have implications for considering that they are at different stages of structural transformation.

In addition, in lines 341-342, it says that “We pay specific attention to education, sector and occupation, as structural transformation is typically associated with changes in these dimensions.” Looking at the tables, it can be seen that there are differences between the levels of education in the countries, although not very pronounced, and that there are hardly any differences between the distribution of employment by occupations and by sectors across countries. Therefore, one could infer that there are not so many differences in the stage of structural transformation.	In this respect, one wonders about the importance of oil exports in the Nigerian GDP per capita, and how this could distort the feeling that Nigeria is in a more advanced stage than the other two countries.

All this preamble is to try to say that I don't quite understand the relationship between the stage of structural transformation and that the gender wage gap should decrease sooner in Nigeria than in other countries. In fact, authors say (lines 617-619) “Thus, the gender pay gap is not always lower in urban areas or in countries that are at a more advanced stage of structural change. This implies that structural transformation does not consistently help bridge the gender pay gap.” This is also the case in European countries, for example. The Nordic countries have a more advanced stage than those of the South and, nevertheless, the gender wage gap is not less. For example, due to the strong occupational segregation in the Nordic countries.

**Response:** The link between structural transformation and the gender pay gap is not clear ex-ante, as such we do not necessarily expect that the gender pay gap will shrink as structural transformation advances. This is stated in the abstract, and is discussed in the third and fourth paragraphs of the introduction, where we identify potential factors that can reduce or exacerbate the gender pay gap. The aim of our study is to provide empirical evidence of the gender pay gap in non-farm employment in urban and rural areas in Nigeria, Malawi and Tanzania, and to describe possible linkages with structural transformation. As the reviewer noticed, we indeed do not find that the gap is always lower in urban areas or in Nigeria.

Furthermore, we followed the reviewer’s suggestions and made the following changes to the text:

We changed the text in the Introduction to clarify that Nigeria was already at a more ‘advanced’ stage of structural transformation in 2000 – see the italicized text below:

“First, according to several World Development Indicators [39], Nigeria tends to be at a more advanced stage of structural transformation than Tanzania, followed by Malawi. For example, gross domestic product (GDP) per capita in 2019 was 5,348 int.$ in Nigeria, 2,771 int.$ in Tanzania and 1,104 int.$ in Malawi. Non-agricultural employment accounted for a higher share of total employment in Nigeria (65%), compared to Tanzania (35%) and Malawi (24%). Also urbanization rates in 2019 were highest in Nigeria (51%), followed by Tanzania (35%) and Malawi (17%). *All three countries have embarked on the path of structural transformation during the past 20 years.* *While these indicators were also higher for Nigeria in 2000 with similar initial lower levels for Tanzania and Malawi, growth rates have been strongest in Tanzania and lowest in Malawi.”*

We added a couple of sentences to the Discussion to explain the lack of a shift in employment towards higher productivity service sectors – see the italicized text below:

“Considering sectors, there are some differences between the countries in terms of the sectoral distribution of employment, but no clear sign of employment shifting to higher productivity service sectors (e.g., finance/real estate, other services) in Nigeria. *This is in line with other studies that have observed a shift towards non-tradable services in SSA, as opposed to other developing regions [20]. The lack of a shift in employment in Nigeria might be particularly explained by the country’s high reliance on oil exports.* In addition, sectoral segregation by gender and its contribution to the gender pay gap is quite comparable across the three countries.”

A second, less important point, regards to the choice of selection variables, the authors acknowledge that selected variables may not be “perfectly exogenous” to hourly pay estimates. However, the authors maintain their estimates and try to justify them by arguing that according to Wald tests these variables cannot be rejected in the specification. Additionally, they use recentered influence functions as a robustness of their results. Could the authors present some tests of exogeneity and validity of the instruments, as an alternative?

**Response:** We used a Heckman selection model, as opposed to an instrumental variable (IV) model), to account for potential selection bias into non-farm employment – feeding into the subsequent modelling of the gender gaps in non-farm earnings. This was one of our robustness checks. It is not possible to test the exogeneity of the selection variables in a Heckman model (unlike an IV model), and the assumption regarding their exogeneity in this context is rooted in the cited literature that have relied on these variables in comparable analyses of earnings. The Wald tests are used to check for their “relevance” in the process that underlies selection into non-farm employment (i.e., whether they are statistically significant predictors of non-farm employment and cannot be jointly excluded from the first stage probit selection equation).

Minor comment:

Regarding occupations, would it not make sense to include the classification in terms of the one-digit 10 groups of the ISCO classification and not between self-employed and salaried?

**Response:** When “occupation” is classified using the 10 ISCO groups, the decomposition methods do not perform well. A requirement for the decomposition method is overlapping support, which implies that none of the groups of workers is perfectly identified by the vector of observable factors X. We encountered this problem in our regression models when the occupations are specified using the 10 ISCO groups and when the other job and worker characteristics are included. Hence, we opted to specify five groups instead: 1) self-employed with family labor, 2) self-employed without family labor, 3) low-skilled wage employed, 4) medium-skilled wage employed, and 5) high-skilled wage employed.

The distinction in wage employees is based on the ISCO classification and the ILO definitions, with low-skilled being the elementary occupations; medium-skilled the clerical support workers, service and sales workers, skilled agricultural, forestry and fishery workers, craft and related trades workers, plant and machine operators and assemblers, and armed forces; and high-skilled the managers, professionals, technicians and associate professionals.

We clarified this in the revised manuscript – see the italicized text below:

“Occupation is classified according to the International Standard Classification of Occupation (ISCO) and includes dummies for self-employment with household workers (irrespective of the duration and payment of these workers), low-skilled wage employment *(corresponding to elementary occupations)*, medium-skilled wage employment (*corresponding to clerical support workers, service and sales workers, skilled agricultural, forestry and fishery workers, craft and related trades workers, plant and machine operators and assemblers, and armed forces)* and high-skilled wage employment *(corresponding to managers, professionals, technicians and associate professionals)* with self-employment without household workers as base level.

**Reviewer #2:**

All comments have been addressed.
